# A New Treatment Approach for Acute Propanil Poisoning: A Case Report

**DOI:** 10.7759/cureus.26416

**Published:** 2022-06-29

**Authors:** Vaishnavi Arunpriyandan, Sundaresan KT, Maheswaran Umakanth

**Affiliations:** 1 General Medicine, University Medical Unit, Teaching Hospital Batticaloa, Batticaloa, LKA; 2 Clinical Medicine, Faculty of Health Care Sciences, Teaching Hospital Batticaloa, Batticaloa, LKA; 3 Clinical Medicine, Eastern University, Sri Lanka, Chenkalady, LKA

**Keywords:** propanil induced haemolysis, propanil induced acidosis, sri lanka, low resource setting, blood transfusion, venesection, methylene blue, exchange transfusion, methemoglobinemia, propanil

## Abstract

Propanil is a widely used herbicide in agriculture and is also an important cause of poisoning in Sri Lanka. Incidence is around 2% and is commonly reported as self-poisoning. Although it is classified as an agent with low to medium toxicity, severe poisoning can cause lethal outcome and death especially when there is a limited medical facility. We describe a case of severe Propanil poisoning who was successfully treated in a peripheral hospital with available facilities.

## Introduction

Acute propanil poisoning is a significant cause of death among pesticide poisoning. It is the most lethal herbicide poisoning next to paraquat in Sri Lanka. The mortality varies from 6.2 to 10.7% depending on the severity of the poisoning [1,2]. Methemoglobinemia, hemolysis, and neurological sequel at admission predict poor prognosis [3]. Methylene blue is the recommended treatment modality, but there are instances where advanced management options like exchange transfusion are required in order to prevent death [1]. It can be challenging in health care with resource-poor settings.

## Case presentation

**Figure 1:**
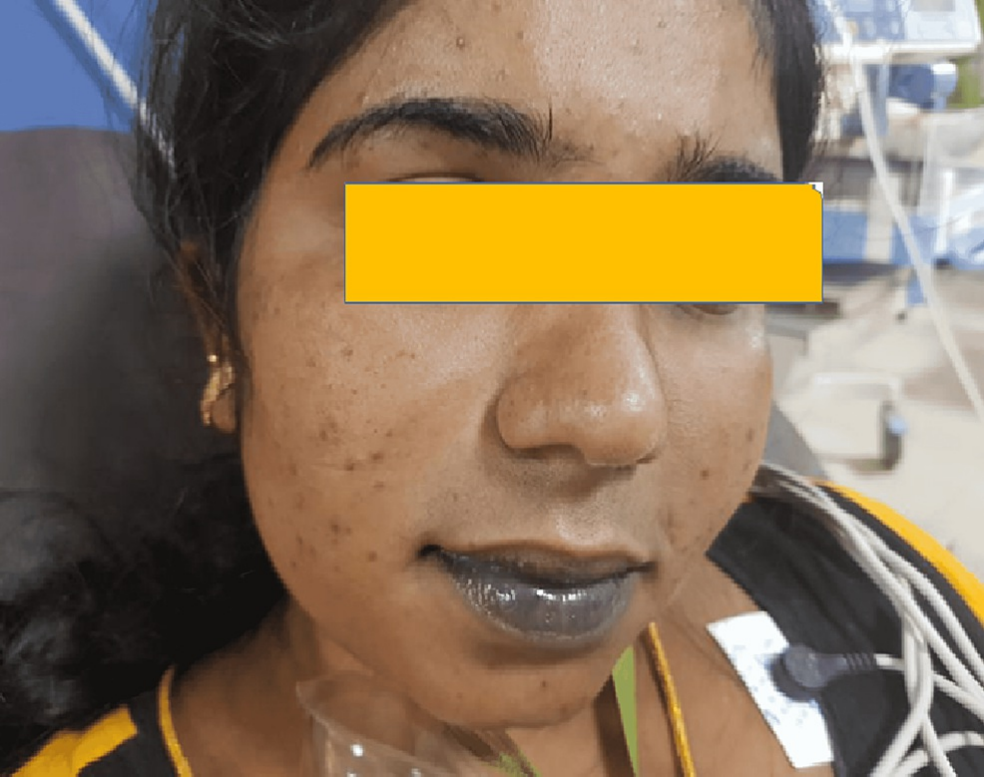
Central cyanosis following acute propanil poisoning

A 24-year-old Sri Lankan lady from a remote area of Sri Lanka presented three hours following the self-intake of nearly 300ml of the liquid form of propanil (concentration- 360g/L) to the emergency room. She was drowsy with severe central and peripheral cyanosis (Figure 1).

**Figure 2:**
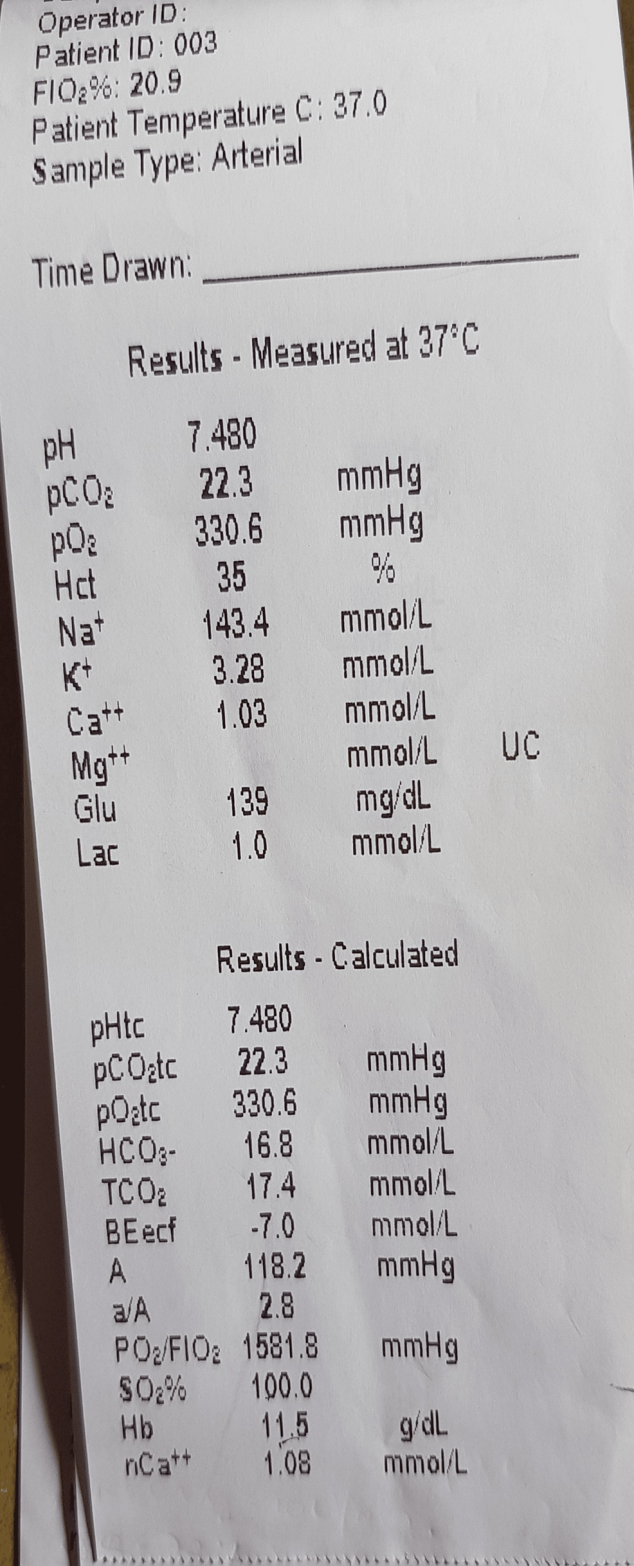
Arterial blood gas analysis showing very high partial pressure of oxygen and a normal oxygen saturation due to the presence of methemoglobin.

Her Glasgow Coma Scale (GCS) was 12/15, respiratory rate was 20/min, and peripheral saturation was 77% on room air with a pulse-oximetry. The arterial blood gas analysis was as follows: pH - 7.24, HCO3 − -17 mmol/l, pCO2 - 28 mmHg, pO2 - 209 mmHg and oxygen saturation - 100% (Figure 2). Repeated bolus of intravenous methylene blue was initiated with close monitoring saturation and arterial blood gas. The color chart for detection of methemoglobin level is used, as there was no facility to do arterial methemoglobin level. She showed only mild improvement in oxygen saturation after an accumulated dose of 700mg of methylene blue. The patient’s saturation was persistently 62% at 48 hours of admission with tachycardia of 132 beats per minute. Measures taken to transfer the patient to a tertiary center with an exchange transfusion facility failed, due to the unavailability of intensive care unit beds.

Therefore, after considering local research and past experience, a decision was made to start cycles of venesection followed by blood transfusion, which had been tried previously at poor resource settings in Sri Lanka with a successful outcome. Accordingly in each cycle, around 450ml of blood was removed via venesection and an equivalent amount of fresh blood was transfused soon after. The patient was vigilantly monitored throughout the procedure. The pulse oximetry saturation increased by 8% with each cycle and finally became normal after three cycles of venesection followed by blood transfusion. She also improved clinically and was discharged on Day 8 of admission after counselling. She did reasonably well on her subsequent follow-up.

## Discussion

Propanil is a very potent herbicide from the acetanilide group. It is sold under different brands mainly in the form of 36% solutions [1]. It is commonly described as a poison with mild toxicity in toxicological literature [3]. The presented case shows that a severe form of poisoning is possible, particularly when taken in large quantities. The lethal dose is around 10ml in undiluted compound. More than 200ml is considered as severe poisoning in diluted propanil [4]. Although the elimination half-life of propanil is approximately 3.2 hours, it can be variable due to the persistent presence of a higher concentration of its main metabolite, 3,4-dichloroaniline.

3,4-dichloroaniline has a longer half-life and is also relatively less lipophilic than propanil, which makes its distribution smaller [2]. Although symptoms of poisoning occur soon after ingestion, the time to death is usually greater than 24 hours, which gives more possibilities for interventions that prevent death [3]. The onset of the development of methemoglobinemia correlates with the level of toxicity.

Clinical toxicity is characterized by cyanosis, hypotension, acidosis and progressive end-organ dysfunction which are compatible with severe and prolonged methemoglobinemia [3]. Methemoglobinemia occurs as a result of bioconversion of propanil to 3,4-dichlorophenyl)hydroxylamine, which is co-oxidized with oxyhemoglobin in erythrocytes to the ferric state [5]. Hemolysis and hepatitis are the other less common manifestations that occur in nearly 1/3rd of the patients, possibly due to the direct oxidant damage to the RBC [6].

Our patient also presented with a rapid onset of methemoglobinemia evidenced by cyanosis, altered consciousness and low oxygen saturation which is possibly due to the rapid production of methemoglobin, due to a large amount of propanil ingestion.

Intravenous methylene blue at doses of 1-2 mg/kg, is the drug of choice in methemoglobinemia. It acts by increasing the rate of conversion of methemoglobin to hemoglobin [4]. However, it might not be adequate in severe poisoning, as in our patient who deteriorated, despite repeated doses of methylene blue. Such patients might require advanced therapeutic options such as Exchange transfusion, which replaces methemoglobin as well as eliminate the poison in the body [4].

The drawbacks are that the efficiency may reduce with the amount of poison situated outside the vascular compartment, which is higher in propanil due to its lipophilic nature leading to a high volume of distribution [2]. However, Exchange transfusion can be lifesaving as it instantly provides fresh erythrocytes, which finally enhances the oxygenation and prevent end-organ damage [1]. Therefore, in case of severe poisoning, we recommend the early transfer of patients to tertiary care hospitals where exchange transfusion facilities are available. Yet there are incidences where an urgent transfer is not possible as in our patient.

Hence a need for an alternative measure was raised, in order to save the life of the patient. Therefore, we thought of a method that can provide equivalent results of exchange transfusion, which is replenishing methemoglobin with normal RBC. Here, we used venesection as a measure of removal methemoglobinated RBC, followed by blood transfusion, as a method of provision of healthy RBCs. Our patient’s condition improved dramatically with each of the venesection-blood transfusion cycle.

Considering the rapid improvement in our patient, we think venesection-blood transfusion cycles may be lifesaving, in cases of severe propanil poisoning in a poor resource setting, where facilities for exchange transfusion or transfer to such institutes are not available. Although not reported, this method has been tried in the remote settings in Sri Lanka, with a fairly good success rate. A good example of it is Pollannaruwa, another rural area of Sri Lanka where propanil poisoning is fairly common.

In addition, studies conducted in Sri Lanka have found difficulties in the management of propanil poisoning due to a lack of methylene blue IV, to measure levels of methemoglobin and beds in intensive care units in small regional hospitals. Also, exchange transfusion facilities are not readily available in these hospitals [1,4,6].

Although the alternative measure was successful, we foresee the importance of further research, to establish treatment modalities that are useful in resource-poor settings.

## Conclusions

Propanil can cause severe clinical manifestations, when taken in large amounts. Despite it being categorized as a mild toxicity compound, it can be potentially lethal without appropriate treatment such as intravenous methylene blue and exchange transfusion. Since facilities of exchange transfusion are not available or not readily accessible in the local hospitals, which face more cases of acute propanil poisoning, early transfer of patients to tertiary care hospitals should be considered.

Alternative treatment modalities are required when a timely transfer is not possible, which is not an uncommon scenario in countries like Sri Lanka. We used venesection-blood transfusion cycles as a life-saving measure in this patient. However further studies on a large population are required to decide on the timing and efficacy of such treatment modalities.
